# Barriers and facilitators to physical activity in people with an inflammatory joint disease: a mixed methods study

**DOI:** 10.1186/s12891-022-05847-z

**Published:** 2022-10-05

**Authors:** Kirsty Bell, Monserrat Conde, Gordon Hendry, Danny Rafferty, Martijn Steultjens

**Affiliations:** 1 NHS Tayside, Physiotherapy Department Crieff Community Hospital, King Street, Crieff, PH7 3HR UK; 2 Centre for Evidence Based Medicine, Nuffield Department of Primary Care Health Sciences, University of Oxford, Oxford, UK; 3 Institute of Health Research, Glasgow Caledonian University, Cowcaddens Road, Glasgow, G4 0BA UK

**Keywords:** Exercise, Physical activity, Inflammatory joint disease, Barriers, Facilitators, Mixed methods

## Abstract

**Background:**

Physical activity has been shown to be of great benefit to people with an inflammatory joint disease (IJD), however people with an IJD have been shown to be very inactive compared to the general population. The aims of this study were to explore 1) whether the transition from a National Health Service (NHS)-run exercise programme into exercising in the community could be achieved successfully; and 2) the barriers and facilitators during the transition period.

**Methods:**

This study adopted a complementary mixed-methods study design including a qualitative approach using focus groups and a prospective cohort study. Descriptive statistics were used to summarise the cohort study data. All variables were assessed for normality of distribution using the Sharpiro-Wilk test. Paired t-tests or Wilcoxon tests were undertaken for two consecutive assessment timepoints; one-way repeated measures ANOVAs or Friedman’s tests for three consecutive assessment timepoints. Micro-interlocutor analysis was used to analyse the focus group data. Areas of congruence and incongruence were explored by confirming the statistical results against the qualitative results. The adapted ecological model of the determinants of physical activity was then used as a framework to describe the findings.

**Results:**

A successful transition was defined as still exercising in the community 6-months post discharge from the NHS-run Inflammatory Arthritis Exercise Programme. This was self-reported to be 90% of the cohort. An individual barrier to physical activity in people with an IJD was found to be the unpredictable nature of their condition. Other barriers and facilitators found were similar to those found in the general population such as recreation facilities, locations, transportation and cost. Other facilitators were similar to those found in people living with other chronic long-term conditions such as the importance of peer support.

**Conclusions:**

90% of the cohort data were defined as a successful transition. People with an IJD have similar barriers and facilitators to exercise as the general population and those living with other chronic long-term conditions. A barrier which appears to be unique to this population group is that of the unpredictable nature of their condition which needs to be considered whenever tailoring any intervention.

**Supplementary Information:**

The online version contains supplementary material available at 10.1186/s12891-022-05847-z.

## Introduction

Physical activity has been shown to be of great benefit to people with an inflammatory joint disease (IJD). It improves joint health, physical function and quality of life [1–3]. It has also been shown to reduce disease activity, reverse rheumatoid cachexia and reduce the risks of cardiovascular disease (CVD), which is higher in the RA population compared to the general population [4–10]. More importantly research has demonstrated that physical activity has no detrimental effects such as joint damage, an increase in disease activity or joint pain [1, 2]. A recent review of physical activity in people with an IJD suggests that the physical activity recommendations from the American College of Sports Medicine; are effective, feasible and safe for people with an IJD [3] and should be an integral part of standard care for people with these conditions.

Despite these health benefits and evidence regarding the effectiveness, feasibility and safety of the physical activity recommendations, people with an IJD have been shown to be very inactive compared to the general population [4]. Emerging evidence is also showing that people with Rheumatoid Arthritis (RA) are not just inactive but spend a lot of time in sedentary behaviours [11], defined as an energy expenditure ≤1.5 METs in a sitting or reclining posture [12]. This has been shown to bring health risks independently associated with inactivity such as increasing risk further for developing CVD, cancer, stroke and diabetes [13]. Lack of physical activity has been explained by both disease specific and personal factors such as pain, fatigue, lack of belief in its benefits, lack of motivation and low self-efficacy [14, 15]. However, there is also evidence to suggest that people with an IJD are aware of the health benefits yet are still found to be less active than the general population [16, 17].

The Transtheoretical Model (TTM) has been used as a framework to investigate the barriers, benefits and preferences for exercise in people with RA [14]. The TTM posits that health behaviour change involves progress through six stages of change: precontemplation, contemplation, preparation, action, maintenance, and termination. Research conducted in this area has suggested that people’s behaviour towards exercise is improved more efficiently if interventions are matched to the individual’s stages of change [18]. People with RA were mainly found to be in pre-contemplation and maintenance stages, each requiring different needs in terms of exercise advice and support. Research in Scandinavia suggests that people with RA prefer to be advised on physical activity by a rheumatologist or a specialist in exercise and RA [14]. They also found that people who were less active exhibited a higher body mass index (BMI), were more likely to be unemployed, have lower quality of life scores and report fewer exercise benefits and more barriers to exercise [14]. This highlights the importance of the benefits of exercise being emphasised to people with an IJD and the need for identification of barriers such as a lack of advice, information and referrals to exercise facilities being addressed by Health Professionals [14, 19].

There is evidence to suggest that the advice and information given on physical activity for people with an IJD from Health Professionals can be inconsistent; with a lack of clear content, continuity and guidance [17]. It appears that there may be a lack of exercise knowledge amongst Health Professionals with regards to frequency, intensity, type and time that should be recommended to people with an IJD. This poses a significant challenge to people with an IJD undertaking physical activity, especially those who are inactive and spend a lot of time sedentary. It has also been found that people with an IJD largely will not initiate discussions about physical activity unless the issue is raised by their consultant [17]. However, there are often significant time demands and competing priorities in routine rheumatology consultations and physical activity discussions are often overlooked. This highlights how important Health Professionals’ roles may be in encouraging people with an IJD to undertake physical activity [14, 19], and emphasising the benefits of exercise.

Despite this, exercise therapy in one form or another is a common intervention for people with an IJD and could potentially provide a steppingstone to remaining physically active. There has been very little research to date looking into what the barriers and facilitators may be to physical activity following completion of exercise therapy.

The Social Ecological Model has been used in the general population as a framework to investigate physical activity due to its multilevel comprehensive approach to the possible determinants but does not appear to have been utilised in an IJD population. The model takes a broad view of health behaviour causation, with the social and physical environment included as contributors to physical activity, particularly those outside of the health sector, such as the recreational facilities available, accessibility, affordability and safety [20].

Understanding why people are physically active or inactive in a broader context is important as it can aid planning of public health interventions. Using the ecological model, this study sought to investigate how easy or difficult it is to make the transition from a Health Professional-led disease-specific exercise programme within the United Kingdom (UK) National Health Service (NHS), into a community exercise setting. Specific aims of this study were to explore 1) whether the transition from an NHS-run exercise programme into exercising in the community could be achieved successfully; and 2) the barriers and facilitators during the transition period.

## Methods

### Design

This study adopted a complementary mixed-methods study design including a qualitative approach using focus groups and a prospective cohort study. Both study components were approved by the National Health Service (NHS) Health Research Authority, NRES Committee South West – Exeter, UK [Ref: 14/SW/1183]. All participants provided written informed consent and the study was undertaken in accordance with the Declaration of Helsinki.

### Participants

**Fig. 1:**
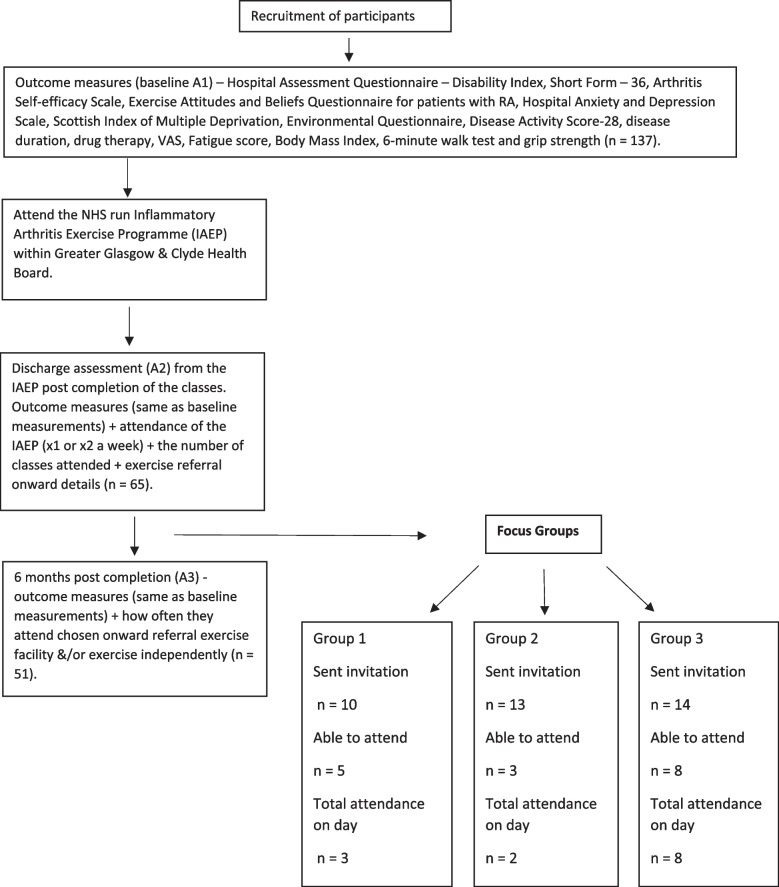
Participant flow diagram detailing the recruitment of patients and data collection

Patients were recruited into the prospective cohort study from referrals into a NHS-run Inflammatory Arthritis Exercise Programme (IAEP) across Greater Glasgow & Clyde (GG&C) Health Board. The NHS is a nation-wide universal health care system in Britain which is free at the point of provision. GG&C Health Board is the largest Health Board in Scotland serving 1.2 million people with wide and variable socioeconomic characteristics. The IAEP is a 12-week exercise programme run by rheumatology physiotherapists across GG&C Health Board. Any adult within the Health Board who has a clinician confirmed IJD and is under the care of the Rheumatology Department can be referred into the programme. The prospective cohort study involved collection of data at 3 key time points: prior to commencing the IAEP (baseline) – assessment 1 (A1), post completion of the IAEP (12 weeks from baseline) – assessment 2 (A2), and 9 months from baseline – assessment 3 (A3). Participants were recruited into the focus groups from the prospective cohort study between A2 and A3 – see Fig. 1.

### Inclusion criteria

Patients referred into the IAEP were included in the study if they met all of the following inclusion criteria: 1) physician-confirmed diagnosis of IJD such as Rheumatoid Arthritis, Psoriatic Arthritis, Ankylosing Spondylitis or any other type of inflammatory arthritis/polyarthritis, 2) were aged 18 years or over.

### Exclusion criteria

Patients were excluded from the study if they met any of the following criteria: 1) did not provide informed consent to be part of the study, 2) were unable to complete the study within the designated data collection period, 3) the presence of co-morbidity severely limiting the patient’s ability to participate in an exercise programme such as unstable angina, heart failure, uncontrolled heart arrhythmias, uncontrolled hypertension, severe respiratory condition, uncontrolled epilepsy, uncontrolled diabetes, recent medical instability such as a stroke, wheelchair user and pregnancy.

### Recruitment strategy

Sampling was undertaken by convenience through identification of eligible participants from consecutive referrals. The sampling frame was limited to the study population of interest which comprised of patients who were under the care of the Rheumatology Department across GG&C Health Board and who were referred into the IAEP between March 2015 to July 2017. Referrals into this programme were made by rheumatology consultants, rheumatology nurse specialists, rheumatology allied health professionals and patients via self-referral. Every patient who was referred into this programme and met the inclusion/exclusion criteria for the study was informed in writing and verbally of the research project by their rheumatology specialist physiotherapist at a screening appointment prior to attending the programme. If the patient was interested in being part of the study, they were subsequently contacted by the researcher for further information. Once willingness was confirmed, participants were booked in for their baseline session where written informed consent was obtained.

### Prospective cohort study data collection

Data was collected by the researcher (KB) at each study time point (see Fig. 1). Health related quality of life (HRQoL) was measured using the Short Form – 36 (SF36) and Hospital Assessment Questionnaire – Disability Index (HAQ-DI); self-perceived levels of control were measured using the Arthritis Self-efficacy Scale (ASES); attitudes and beliefs towards physical activity were measured using the Exercise Attitudes and Beliefs Questionnaire for patients with RA (RA-EAQ); and mental health was measured using the Hospital Anxiety and Depression Scale (HADS), all of which have good psychometric properties which have been verified in populations with IJD [21–25]. The Scottish Index of Multiple Deprivation (SIMD) is a composite measure of social deprivation which has seven domains: current income, employment, health, education, skills and training, housing, geographic access and crime. These seven domains are calculated and weighted for small areas, called ‘data zones’, with roughly equal population and can be obtained using participant postcodes [26].

The Disease Activity Score (DAS-28) was recorded as a marker of disease activity by the researcher who was trained in undertaking the DAS-28. Acute phase reactants from blood test results (within 3 months of each data collection session) were obtained from the patient’s medical records to complete the DAS-28 score. Disease duration was measured from the date of physician-confirmed diagnosis which was obtained from the participant’s medical records. Drug therapy was obtained from the patient’s medical records and clarified with the patient in case of any recent changes; the level of pain on average over the past week was measured using a pain visual analogue scale (VAS) and the level of fatigue was measured using a 100 mm fatigue VAS [8, 27].

To evaluate whether there are any physical-condition-related and/or environmental factors that could determine physical activity levels and sedentary behaviour the following measurements were undertaken. Body Mass Index (BMI); 6-minute walk test [9, 28–30]; grip strength using a JAMAR grip dynamometer and the Southampton protocol [9, 28, 31]; and a custom-made environmental questionnaire to elicit information concerning attendance to an exercise facility or exercising independently, cost, affordability, transportation to/from and the variety of activities on offer at the community exercise facilities. A successful transition could be determined from this questionnaire which was defined as still exercising in the community 6-months post discharge from the NHS-run IAEP. This questionnaire was developed with assistance from the study Advisory Board which consisted of rheumatology clinicians, NHS health improvement officers, patients and academics.

#### Focus group methods

Topics for discussion were developed with assistance from the study Advisory Board which consisted of rheumatology clinicians, NHS health improvement officers, patients and academics. Topics were attitudes towards exercise, beliefs about the impact of exercise on their disease, other personal factors that can act as barriers or facilitators towards sustained healthy exercise behaviour and environmental factors. They discussed how these attitudes and beliefs have changed by participating in the IAEP and how they are self-managing in the community. Three focus groups were conducted with patients who were recruited using purposive convenience sampling from the prospective cohort study. The researcher (KB) lead the semi-structured focus groups with an assistant (MC) who recorded level of consensus using a focus group consensus matrix [32] (supplementary material). Both researchers were physiotherapists who had undertaken training in qualitative research. All focus groups were recorded using a digital voice recorder and recordings were transcribed verbatim.

### Analysis

Descriptive statistics were used to summarise the cohort study data. All variables were then assessed for normality of distribution using the Sharpiro-Wilk test. Paired t-tests or Wilcoxon tests were undertaken for two consecutive assessment timepoints; one-way repeated measures ANOVAs or Friedman’s tests for three consecutive assessment timepoints. Data analysis was undertaken using IBM SPSS version 26 and statistical significance level was *p* < 0.05. Three focus groups were undertaken. The same themes ran through all the focus groups suggesting theoretical data saturation was reached. Micro-interlocutor analysis [32] was used to analyse the focus group data which included thematic analysis of the focus group transcriptions with additional analysis of the matrix for assessing the level of consensus within the focus groups. This enabled group dynamics to be included in the data analysis which increases scientific rigour of focus group analysis [32]. To further enhance scientific rigour two researchers (KB, MC) independently analysed the transcripts using thematic analysis to confirm emerging themes [33, 34]. A final discussion was conducted between the researchers where the data from the three focus groups were integrated, discussed and clarified using Micro-interlocutor analysis [32]. After completing the quantitative and qualitative analyses independently, data from both sets were linked for a more robust understanding of findings. Areas of congruence and incongruence were explored by confirming the statistical results against the qualitative results. The adapted ecological model of the determinants of physical activity [20] was then used as a framework to describe the findings from both the quantitative and qualitative analysis. The framework has 5 main categories: individual, interpersonal, environment, regional or national policy and global. This framework has been used to describe the determinants of physical activity in adults and children across the world [20].

## Results

Figure 3 shows that the majority of participant’s condition are well controlled or are in remission. However, the scores do fluctuate across the 3 different timepoints for all participants. They appear to decrease whilst participants are attending the NHS-run inflammatory arthritis exercise programme, which is between assessment 1 and 2. They then appear to increase between assessment 2 and 3 which is when participants are trying to remain active independently in the community following transition from an NHS-run programme.

Figure 4 shows that pain scores across the 3 different assessment timepoints fluctuate both up and down for all participants.

Figure 5 shows that fatigue scores across the 3 different assessment timepoints fluctuate both up and down for all participants.

Figure 6 shows that self-efficacy improves for the majority of participants whilst attending the NHS-run inflammatory arthritis exercise programme (A1- A2) however decreases slightly following transition into the community (A2 -A3). The majority of scores at assessment 3 appear to be higher than those assessment 1. Also, the majority of participants had fairly high self-efficacy scores at assessment 1.

Figure 7 shows that exercise attitudes and beliefs improve for the majority of participants whilst attending the NHS-run inflammatory arthritis exercise programme (A1- A2). However, vary following transition into the community (A2 -A3). Some appear to level off, some increase and some decrease. The majority of scores at assessment 3 appear to be higher than those at assessment 1. Also, the majority of participants appear to have good attitudes and beliefs towards physical activity at assessment 1.

Table 3 shows that when the cohort data for the participants in the focus groups was statistically analysed there was a significant difference in DAS-28 and pain scores between assessment 1 & 2, however not between assessment 2 & 3. Fatigue scores across assessment 1 & 2 were close to there being a significant statistical difference. There was found to be no significant statistical difference across the 3 assessment timepoints for DAS-28, pain or fatigue scores. There was also found to be no significant difference in ASES and RA-EAQ scores between assessment 1 & 2, assessment 2 & 3 and across the 3 assessment timepoints.

## Discussion

A successful transition was defined as still exercising in the community 6-months post discharge from the NHS-run IAEP. This was self-reported to be 90% (46) of the cohort (Table 2). Table 1 shows that 49% (25) of the cohort study were referred onto local Government-run (Council-run) exercise facilities and 51% (26) onto independent exercise facilities/activities from the NHS-run IAEP. It does raise a question as to why everyone was not referred onto an exercise facility from the IAEP, as Bell et al. [35] found that people with an IJD who attend an exercise facility in the community are more physically active than those who independently exercise. This may have been due to patient choice or as a result of a busy clinic with competing time priorities and a referral may never have been made. Further research into this would need to be undertaken. Table 2 shows that at assessment 3, which is 6 months post discharge from the NHS-run IAEP, 43% (22) were attending an exercise facility, 47% (24) were exercising independently and 10% (5) were not exercising. The barriers and facilitators to achieving this will be discussed below.

**Table 1:** Participant Demographics

	Focus Groups (*n* = 13)	Cohort Group (*n* = 51)
Female, n (%)	10 (77%)	41 (80%)
Mean Age (SD)	64.54 (9.3)	61.86 (9.57)
Mean Disease Duration (SD)	10.08 (13.62)	10.65 (10.54)
SIMD quintiles (1 most exposure to deprivation, 5 least exposure to deprivation), n (%)	1–1 (8%)	1–9 (18%)
	2–1 (8%)	2–12 (23%)
	3–2 (15%)	3–4 (8%)
	4–4 (31%)	4–12 (23%)
	5–5 (38%)	5–14 (28%)
Referral onwards to local government-run (council-run) exercise facilities post NHS-run IAEP, n (%)	6 (46%)	25 (49%)
Referral onwards to independent exercise facilities/activities post NHS-run IAEP, n (%)	7 (54%)	26 (51%)

**Table 2:** Self reported exercise levels at assessment 1 (A1), assessment 2 (A2) and assessment 3 (A3)

	Attend Exercise Facility	Independent Exercise	Not Exercising
Cohort A1 (*n* = 51) n (%)	17 (33%)	27 (53%)	7 (14%)
Focus Group A1 (*n* = 13) n (%)	5 (38%)	7 (54%)	1 (8%)
Cohort A2 (*n* = 51) n (%)	18 (35%	29 (57%)	4 (8%)
Focus Group A2 (*n* = 13) n (%)	5 (38%)	7 (54%)	1 (8%)
Cohort A3 (*n* = 51) n (%)	22 (43%)	24 (47%)	5 (10%)
Focus Group A3 (*n* = 10) n (%)	7 (70%)	2 (20%)	1 (10%)

**Fig. 2:**
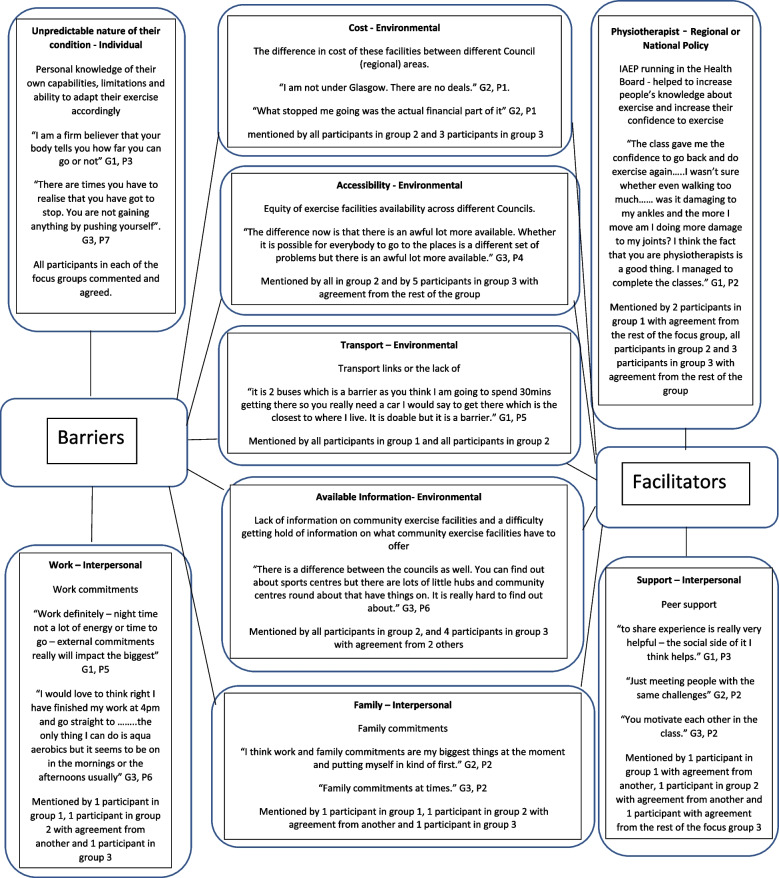
Mapping of the main themes from the focus groups

Participants in the focus groups were a good representation of the cohort group (Table 1). Some of the barriers and facilitators found from the focus group data to remaining physically active following discharge from an NHS-run exercise programme were similar to those found in the general population [20]. Strong correlates to physical activity in the general population have been found to be environmental factors such as recreation facilities, locations and transportation [20] which can also be seen from the focus group data in Fig. 2. These factors are environmental on the Social Ecological Model [20] which are greatly influenced by regional and national policy. Cost was also a barrier to exercising in the community and Fig. 2 highlights the variability in cost of exercise facilities across different regions. Such findings may also highlight the importance that social determinants of health have in physical activity behaviour of people with IJD.

**Fig. 3:**
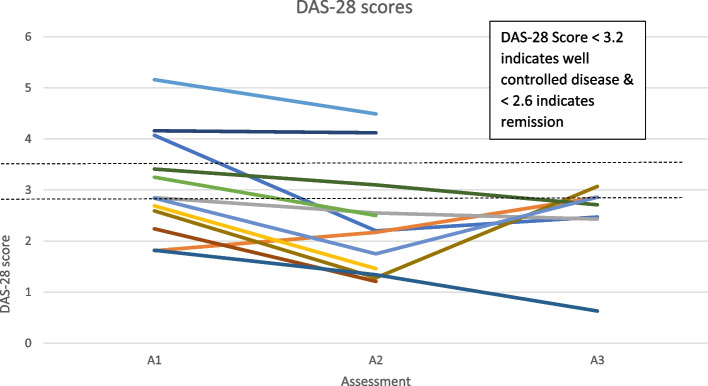
Disease activity scores (DAS-28) for 13 participants in focus groups across 3 assessment time points

**Fig. 4:**
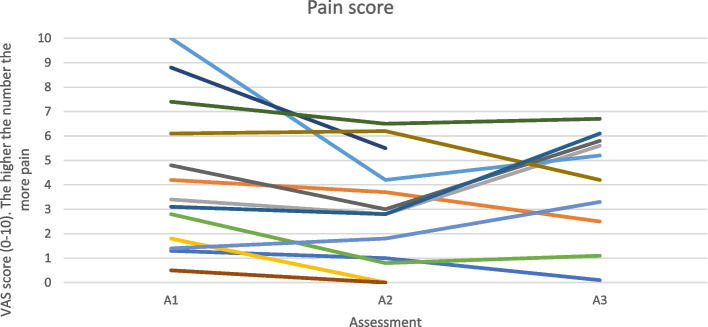
Pain scores for 13 participants in focus groups across 3 assessment time points

**Fig. 5:**
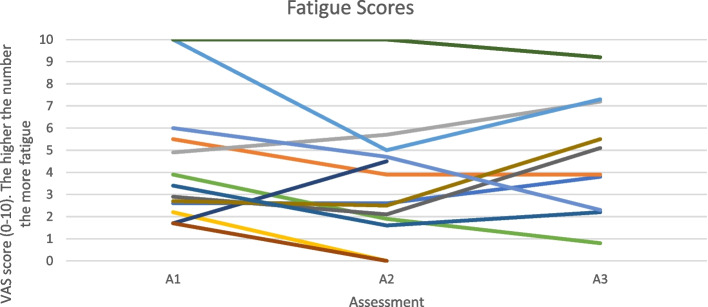
Fatigue scores for 13 participants in focus groups across 3 assessment time points

**Fig. 6:**
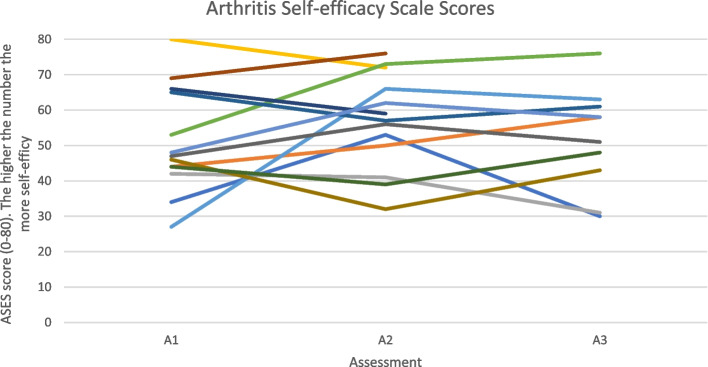
Arthritis Self-efficacy Scale (ASES) scores for 13 participants in focus groups across 3 assessment time points

**Fig. 7:**
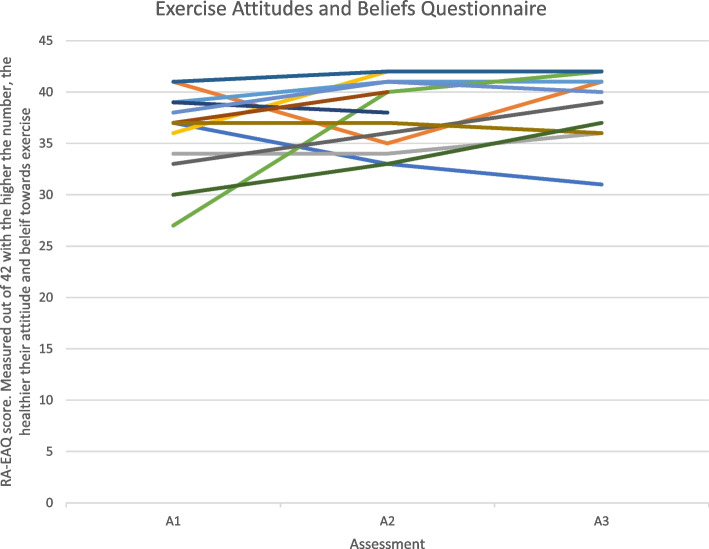
Exercise Attitudes and Beliefs Questionnaire for people with RA (RA-EAQ) scores for 13 participants in focus groups across 3 assessment time points

An individual barrier to physical activity in people with an IJD from the focus group data was found to be the unpredictable nature of their condition. This can be seen from the cohort data in Figs. 3, 4 and 5 by the variable levels of disease specific factors such as pain, fatigue and DAS-28 scores across the 3 assessment timepoints. However, when the focus group cohort data was taken forward for statistical analysis seen in Table 3, the significance levels were variable which could be explained by the small sample size. This therefore appears to correspond with many findings from both quantitative and qualitative research [14, 15, 17, 27, 36, 37] but has never been demonstrated in a mixed methods paper following completion of a NHS-run IAEP. Figure 6 appears to illustrate that self-efficacy increased whilst attending the IAEP (A1 to A2), linking in with the findings of Henchoz et al. that people with an IJD prefer to be advised on physical activity by a specialist in exercise and RA [14] and then looks to decrease slightly following the transition onto exercising in the community (A2 to A3). However, when the focus group cohort data was taken forward for statistical analysis seen in Table 3, there was no significant difference found between the 3 assessment timepoints. Figure 7 appears to correlate with the findings of Rongen-van Dartel et al. and Law et al. that people with an IJD are aware of the health benefits of exercise as the scores of the Exercise Attitudes and Beliefs Questionnaire for patients with RA are very similar across the 3 assessment time points and are fairly high at assessment 1. This can be further seen in Table 3 where no statistical significance difference was found across the assessment timepoints. The barriers found above which if addressed could become facilitators of physical activity also correlate with the recent findings of Davergne et al. [38] that the main major barrier or facilitator to physical activity was related to people’s physical condition such as the presence/absence of symptoms such as pain, fatigue and stiffness. They also found the second main barrier or facilitator was having the knowledge that physical activity is good for health. All of which are modifiable factors that Health Professionals can help to address.

**Table 3:** Analysis of focus group participants cohort data across the 3 assessment time points

	DAS-28	Pain	Fatigue	ASES	RA-EAQ
A1 – A2	Paired t-test *p* = 0.002 Paired difference M = 0.73 (0.62) 95% C.I. Lower: 0.33 Upper: 1.12	Paired t-test *p* = 0.015 Paired difference M = 1.33 (1.68) 95% C.I. Lower: 0.31 Upper:2.35	Wilcoxon *p* = 0.068 Ax1 Md = 3.4 (3.35) Ax2 Md = 2.6 (3.10)	Paired t-test *p* = 0.210 Paired difference M = −5.46 (14.86) 95% C.I. Lower: − 14.45 Upper: 3.53	Paired t-test *p* = 0.194 Paired difference M = − 1.77 (4.64) 95% C.I. Lower: − 4.57 Upper: 1.03
A2 – A3	Paired t-test *p* = 0.301 Paired difference M = − 0.28 (0.88) 95% C.I. Lower: − 1.19 Upper: 0.44	Paired t-test *p* = 0.210 Paired difference M = − 0.78 (1.83) 95% C.I. Lower: − 2.09 Upper: 0.53	Wilcoxon *p* = 0.213 Ax2 Md = 2.6 (3.10) Ax3 Md = 4.5 (4.95)	Paired t-test *p* = 0.766 Paired difference M = 1.00 (10.33) 95% C.I. Lower: −6.34 Upper: 8.39	Paired t-test *p* = 0.140 Paired difference M = − 1.30 (2.54) 95% C.I. Lower: − 3.12 Upper: 0.518
A1-A2-A3	One-way repeated measures ANOVA Wilks’ Lambda *p* = 0.163 A1 M = 2.77 (0.81) A2 M = 2.05 (0.65) A3 M = 2.43 (0.83)	One-way repeated measures ANOVA Wilks’ Lambda *p* = 0.151 A1 M = 4.45 (2.73) A2 M = 3.28 (1.94) A3 M = 4.06 (2.24)	Friedman Test *p* = 0.139 Ax1 Md = 4.4 (4.15) Ax2 Md = 3.25 (3.13) Ax3 Md = 4.5 (4.95)	One-way repeated measures ANOVA Wilks’ Lambda *p* = 0.233 A1 M = 45.00 (10.19) A2 M = 52.90 (12.76) A3 M = 51.90 (14.43)	One-way repeated measures ANOVA Wilks’ Lambda *p* = 0.285 A1 M = 35.70 (4.64) A2 M = 37.20 (3.52) A3 M = 38.50 (3.50)

*ASES* Arthritis Self Self-efficacy Scale

*RA-EAQ* Exercise Attitudes and Beliefs Questionnaire for people with RA

Research to date highlights the importance that Health Professionals have in educating people with an IJD to exercise [14, 19], however research has also shown that this advice can be inconsistent and conflicting [17, 36]. It can be seen from Fig. 2 that if this is addressed on a more regional or national policy bases [20] it can be a facilitator to physical activity in people with an IJD, especially as physical activity guidelines for people with an IJD have been published [3]. Figure 2 also demonstrates that although the unpredictable nature of their condition is a barrier there is great importance on personal knowledge of their own capacities, limitations and ability to manage symptoms and adapt their exercise which they appear to gain from the NHS-run IAEP as seen in the cohort data Fig. 6 A1 and A2 timepoints. However, when the focus group cohort data was taken forward for statistical analysis (Table 3), no significant difference was found. Further research would need to be undertaken in this area.

An interpersonal barrier, from the focus group data, was work commitments stating that they only have so much energy in a day therefore do not have enough to undertake both work and exercise. However, research has shown that exercise can improve fatigue levels [16] suggesting the possible need for education in this area to help address this barrier. Education could be undertaken around people’s perception of exercise as recent research has shown that by breaking up time spend sedentary with light-intensity activity can benefit your health [39]. Difficulty accessing facilities/activities is also highlighted here which as mentioned above is an environmental factor which can be influenced by regional/national policy [20].

An interpersonal facilitator to physical activity in people with an IJD is peer support (Fig. 2). The importance of meeting people with the same challenges, sharing experiences and being able to support/motivate each other with similar conditions has been highlighted. This is in agreement with the broader literature concerning people living with other chronic long-term conditions and has become a key component in self-management programmes [40]. However, the effectiveness of peer support in people living with chronic conditions is unclear due to varying research study designs and definitions of peer support [40].

A limitation of this study could be the purposive convenience sampling from the prospective cohort study for the focus groups. However, the focus group was a good representation of the cohort group. Another limitation of the study could be that both researchers who conducted and analysed the focus group data were physiotherapists. They both had prior knowledge and experience in this research area which could be interpreted as a potential source of bias; yet also could have added more meaning to the research [41]. This is an area which has been acknowledged and extensively discussed in qualitative research with Smith & Noble [42] concluding that “researchers bring to each study their experiences, ideas, prejudices and personal philosophies, which if accounted for in advance of the study, enhance the transparency of possible research bias”. Another limitation of this study could be that it did not look into frequency, intensity, type and time of exercise that participants were undertaking across the assessment timepoints. However subjective methods of data collection of physical activity have been found to be less reliable than objective methods [43] especially in the rheumatoid population [44]. Further research would need to be undertaken in this area.

In conclusion, 90% of the cohort data were defined as a successful transition between an NHS-run IAEP and exercising in the community. People with an IJD have similar barriers and facilitators to exercise as the general population and those living with other chronic long-term conditions. A barrier which appears to be unique to this population group is that of the unpredictable nature of their condition which needs to be considered whenever tailoring any intervention. This is a disease-specific factor which Health Professionals working with people who have an IJD could help address through person-centred approaches to enable them to become more physically active.

## Supplementary Information

**Additional file 1.**

## Data Availability

The datasets during and/or analysed during the current study are available from the corresponding author on reasonable request.
