# Supplementary material for: Barriers and facilitators to physical activity in people with an inflammatory joint disease: a mixed methods study
Source: BMC Musculoskelet Disord. 2022 Oct 5;23:897. doi: 10.1186/s12891-022-05847-z (PMC9533590; doi:10.1186/s12891-022-05847-z)
Supplement: Supplementary file 1 — Additional file 1. [file 12891_2022_5847_MOESM1_ESM.docx]

**Focus Group Key**

**A = Agreement**

**D = Disagreement**

**SA = Provided significant statement or example suggesting agreement**

**SD = Provided significant statement or example suggesting disagreement**

**NR = Did not indicate agreement or disagreement (non-responder)**

1. **Introduction**
2. **Attitudes towards exercise**

| Focus Group Question | Member  1 - | Member 2 – | Member  3 – | Member  4 - | Member  5 - | Member  6 - | Member  7- | Member  8 - | Member  9 - | Member  10 - |
| --- | --- | --- | --- | --- | --- | --- | --- | --- | --- | --- |
| Since taking part in the NHS-run exercise classes, have your attitudes and thoughts towards activity and exercise changed?  Firstly, if they have changed, how have they changed? |  |  |  |  |  |  |  |  |  |  |

1. **Beliefs about the impact of exercise on your disease**

| Focus Group Question | Member  1 - | Member  2 - | Member  3 - | Member  4 - | Member  5 - | Member  6 - | Member  7 - | Member  8 - | Member  9 - | Member  10 - |
| --- | --- | --- | --- | --- | --- | --- | --- | --- | --- | --- |
| Do you feel that activity and exercise is good for your condition? If yes, why? |  |  |  |  |  |  |  |  |  |  |
| Do you feel that activity and exercise is bad for your condition? If yes, why? |  |  |  |  |  |  |  |  |  |  |
| Has this changed since taking part in the exercise classes and if so how do you feel it has changed? |  |  |  |  |  |  |  |  |  |  |
| Focus Group Question | Member  1 - | Member  2 - | Member  3 - | Member  4 - | Member  5 - | Member  6 - | Member  7 - | Member  8 - | Member  9 - | Member  10 - |
| Can you give examples of how activity and exercise has made a positive impact on your condition? |  |  |  |  |  |  |  |  |  |  |
| Can you give examples of how activity and exercise has made a negative impact on your condition? |  |  |  |  |  |  |  |  |  |  |

1. **Personal factors that can act as barriers or facilitators towards sustaining healthy exercise behaviour.**

| Focus Group Question | Member  1 - | Member  2 - | Member  3 - | Member  4 - | Member  5 - | Member  6 - | Member  7 - | Member  8 - | Member  9 - | Member  10 - |
| --- | --- | --- | --- | --- | --- | --- | --- | --- | --- | --- |
| Firstly  Since moving onto exercising in the community from the NHS-run exercise classes, what difficulties and challenges have you found trying to exercise and/or remain active? |  |  |  |  |  |  |  |  |  |  |
| Do you think these difficulties and challenges have changed since taking part in the NHS-run exercise classes? In other words are the difficulties and challenges you face now the same as they were prior to attending the NHS-run exercise classes? |  |  |  |  |  |  |  |  |  |  |

| Focus Group Question | Member  1 - | Member  2 - | Member  3 - | Member  4 - | Member  5 - | Member  6 - | Member  7 - | Member  8 - | Member  9 - | Member  10 - |
| --- | --- | --- | --- | --- | --- | --- | --- | --- | --- | --- |
| Secondly  Since moving onto exercising in the community from the NHS-run exercise classes, what things makes you want to continue to remain active and exercise? |  |  |  |  |  |  |  |  |  |  |
| Have any of these things or reasons changed since taking part in the NHS-run exercise classes? |  |  |  |  |  |  |  |  |  |  |

1. **Environmental Factors**

| Focus Group Question | Member  1 - | Member  2 - | Member  3 - | Member  4 - | Member  5 - | Member  6 - | Member  7 - | Member  8 - | Member  9 - | Member  10 - |
| --- | --- | --- | --- | --- | --- | --- | --- | --- | --- | --- |
| Since moving onto exercising in the community from the NHS-run exercise classes, do you feel there are any environmental factors that make it more difficult for you to continue to exercise such as  **I)** The exercise facilities in your area, for example  variety of activities, exercises offered & appropriate level of classes |  |  |  |  |  |  |  |  |  |  |
| Focus Group Question | Member  1 - | Member  2 - | Member  3 - | Member  4 - | Member  5 - | Member  6 - | Member  7 - | Member  8 - | Member  9 - | Member  10 - |
| **II)** Accessibility to these facilities, for example times of classes, transportation & own personal safety getting to and from the facility |  |  |  |  |  |  |  |  |  |  |
| **III)** What do you think about the cost of exercising in the community? Is this an issue for you? |  |  |  |  |  |  |  |  |  |  |
| Focus Group Question | Member  1 - | Member  2 - | Member  3 - | Member  4 - | Member  5 - | Member  6 - | Member  7 - | Member  8 - | Member  9 - | Member  10 - |
| Since moving onto exercising in the community from the NHS-run IAEP, do you feel there are any environmental factors that help you to continue to exercise such as  **I)** The exercise facilities in your area, for example variety of activities and exercises offered & appropriate level of classes for you |  |  |  |  |  |  |  |  |  |  |
| **II)** Accessibility to these facilities, for example times of classes, transportation & own personal safety. |  |  |  |  |  |  |  |  |  |  |
| **III)** Again what do you think about the cost of exercising in the community? |  |  |  |  |  |  |  |  |  |  |
| Final comments |  |  |  |  |  |  |  |  |  |  |
